# Assessment of pulmonary artery stiffness by multiparametric cardiac magnetic resonance-surrogate for right heart catheterization

**DOI:** 10.3389/fcvm.2023.1200833

**Published:** 2023-08-25

**Authors:** Hermann Körperich, Jan Eckstein, Medhat Atito, Peter Barth, Kai Thorsten Laser, Wolfgang Burchert, Oliver M. Weber, Christian Stehning, Misagh Piran

**Affiliations:** ^1^ Institute for Radiology, Nuclear Medicine and Molecular Imaging, Heart and Diabetes Center North Rhine Westphalia, Ruhr-University of Bochum, Bad Oeynhausen, Germany; ^2^ Clinic for Paediatric Cardiology and Congenital Heart Defects, Heart and Diabetes Center North-Rhine Westphalia, Ruhr-University of Bochum, Bad Oeynhausen, Germany; ^3^ Philips Clinical Science, Hamburg, Germany

**Keywords:** cardiovascular magnetic resonance, pulmonary artery stiffness, pulmonary artery pressure, pulse wave velocity, reference values, right heart catheterization, hemodynamics

## Abstract

**Background:**

Cardiac magnetic resonance (CMR) imaging allows for multiparametric assessment of healthy pulmonary artery (PA) hemodynamics. Gender- and aging-associated PA stiffness and pressure alterations have remained clinically unestablished, however may demonstrate epidemiological differences in disease development. The aim of this study is to evaluate the role of CMR as a surrogate for catheter examinations by providing a comprehensive CMR assessment of sex- and age-related reference values for PA stiffness, flow, and pressure.

**Methods and Results:**

PA hemodynamics were studied between gender and age groups (>/<50 years) using phase-contrast CMR. Corresponding correlation analyses were performed. 179 healthy volunteers with a median age of 32.6 years (range 11.3–68.2) were examined. Males demonstrated increased PA compliance (median [interquartile range] or mean ± standard deviation) (20.8 mm^2^/mmHg [16.6; 25.8] vs. 19.2 ± 7.1 mm^2^/mmHg; *P* < 0.033), higher pulse wave velocity (2.00 m/s [1.35; 2.87] vs. 1.73 m/s [1.19; 2.34]; *P* = 0.018) and a reduced full width half maximum (FWHM) (219 ± 22 ms vs. 235 ± 23 ms; *P* < 0.001) than females. Mean, systolic, diastolic PA pressure and pulmonary proportional pulse pressure were significantly elevated for males compared to females (*P* < 0.001). Older subjects (>50 years) exhibited reduced PA elasticity (41.7% [31.0; 52.9] vs. 66.4% [47.7; 83.0]; *P* < 0.001), reduced PA compliance (15.4 mm^2^/mmHg [12.3; 20.7] vs. 21.3 ± 6.8 mm^2^/mmHg; *P* < 0.001), higher pulse wave velocity (2.59 m/s [1.57; 3.59] vs. 1.76 m/s [1.24; 2.34]; *P* < 0.001) and a reduced FWHM (218 ± 29 ms vs. 231 ± 21 ms; *P* < 0.001) than younger subjects.

**Conclusions:**

Velocity-time profiles are dependent on age and gender. PA stiffness indices deteriorate with age. CMR has potential to serve as a surrogate for right heart catheterization.

## 1. Introduction

Estimation of pulmonary artery (PA) stiffness and blood flow characteristics is complex, and various formulas aim to define it (1). PA wave oscillations alter due to vascular remodelling in pulmonary arterial hypertension (PAH) (2). However, hemodynamic changes may occur even in absence of cardiovascular and respiratory disease. Recent literature assesses the importance of such parameters in healthy volunteers. Per example, several echocardiographic studies emphasized the prognostic importance of PA systolic pressure, which increases with age (3–5) and raises mortality (6). To the best of our knowledge, although recent literature has provided insights into various aspects and parameters of pulmonary artery (PA) stiffness, there has been no conducted assessment using cardiac magnetic resonance (CMR) imaging that comprehensively covers the extensive range of potential variables. Although echocardiography remains a widely available, invaluable tool for raising suspicion of pulmonary hypertension, its compromised ultrasound window for right heart assessment and operator-dependence remain respectable limitations. To date, right-sided cardiac catheterization is the gold standard for examination of pulmonary artery pressure (PAP). However, as it remains an invasive procedure, it is not suitable as a screening mechanism. Additionally, intervention-related complications and measurement errors due to patient position, air bubbles, catheter tube distance and transducer height are not uncommon. In contrast, non-invasive CMR provides a sufficiently good spatial and temporal resolution for accurate evaluation of cardiac function and blood flow characterization (7, 8). Moreover, superior correlation of phase-contrast CMR with right-heart catheterization over ultrasound has been described (9).

Gender-specific differences of cardiac and pulmonary hemodynamics have been poorly defined in most pulmonary flow investigations. Evidence for gender-specific differences in cardiovascular hemodynamics (10) and strain (11, 12) have recently been published. Thus, differences in pulmonary flow characteristics between genders may be assumed. These differences may carry epidemiological significance for gender related disease development.

Non-invasive quantification of average blood velocities using CMR have shown promising results for evaluation of pulmonary pressures and impedance (13). Although an age-dependent increase in PA stiffness of healthy subjects has already been observed in some CMR studies (14, 15), a comprehensive CMR assessment, encompassing the broad spectrum of PA stiffness parameters together with associated cardiac remodelling, is crucial, as these may serve as potential hemodynamic biomarkers for subclinical changes. The present study aims to deliver a comprehensive assessment of the multiparametric adaptions in pulmonary stiffness and velocity-to-time parameters associated with age and gender in a healthy cohort using non-invasive CMR. We hypothesize that multiparametric assessment will demonstrate gender- and age-dependent disparities, particularly in aspects of pulmonary arterial pressure, pulse wave velocity, PA capacitance and velocity-to-time profile characteristics. The findings of this study aim to provide new CMR variables that characterize subclinical vascular remodelling influenced by age and sex.

## 2. Method

### 2.1. Study design

For this prospective single-center, cross-sectional study, 208 volunteers were enrolled through a public call from September 2017 to December 2020. The study was approved by the local ethical review board (Ethikkommission der Medizinischen Fakultät der Ruhr-Universität Bochum, Sitz Ostwestfalen, registration number: 2017-238) and conforms with the Declaration of Helsinki, seventh revision of 2013.

Written consent was given by the participants or legal guardians. Using a preceding questionnaire, routine echocardiography, and/or standard cardiovascular magnetic resonance (CMR) imaging, healthy participants without evidence of cardiovascular disease were selected. Exclusion criteria comprised any personal and familial cardiac history, blood pressure medications, associated risk factors and general contraindications for performing CMR. After explaining the examination procedure, CMR was performed to obtain information on PA stiffness, as well as cardiac functional parameters.

Eighteen volunteers were excluded from the study because they did not meet health criteria (e.g., hypertension). Furthermore, eleven subjects were excluded due to technical limitations or insufficient image quality. Therefore, the final healthy cohort consisted of 179 participants covering six age decades as evenly as possible and taking into account a gender distribution that was also even.

### 2.2. Cardiovascular magnetic resonance

CMR imaging was conducted with a multi-transmit 3 T MRI system (Achieva, Philips Healthcare, Best, The Netherlands; Release 5.3.1/5.6.1) with dStream technology. A dedicated cardiac phased-array coil was used for signal reception. Participants were examined in supine position. A vector electrocardiogram was applied to perform cardiac-triggered acquisitions.

#### 2.2.1. Heart function

Cine steady-state free-precession acquisitions were used for standard 2-chamber, 3-chamber and 4-chamber long axis views. A short-axis stack covering the left and right ventricles and an axial whole-heart cine acquisition were applied to assess ventricular and atrial functional parameters. The following imaging parameters were applied: repetition time/echo time/flip angle = 2.7 ms/1.35 ms/42°, 45 cardiac phases (interpolated, 32 acquired cardiac phases), spatial resolution 1.5 × 1.5 × 8 mm^3^, SENSE-reduction factor of 2, breath-hold periods ≤12 s. Volumetric quantifications and strain analysis were conducted using the CVI42® software package (Circle Cardiovascular Imaging Inc., Calgary, Canada, Release 5.12.1). Global longitudinal, circumferential and radial right ventricular (RV) strain was quantified based on the cine 4-chamber long axis and short axis views. The tricuspid annular plane systolic excursion (TAPSE) was defined as the difference between end-diastolic and end-systolic length measured at the lateral tricuspid annulus-to-apex in the 4-chamber view.

#### 2.2.2. Pulmonary artery stiffness by phase-contrast CMR

Quantitative blood flow measurements in the main PA, ascending aorta (AO), and in the mitral valve plane (for transmitral flow) were done with a conventional flow-encoded pulse sequence. To achieve a high temporal resolution of 10 milliseconds, a TR/TE/flip angle of 10 ms/3.3 ms/30° was applied in combination with a variable number of heart phases based on the subjects's heart rate (i.e., heart rate of 60 bpm corresponds to 100 heart phases). A fixed velocity-encoding value of 200 cm/s was used for arteries and 100 cm/s for transmitral flow. Blood flow analyses were performed using the “HDZ MR-Tools” software package developed at our institution (16).

**Figure 1:**
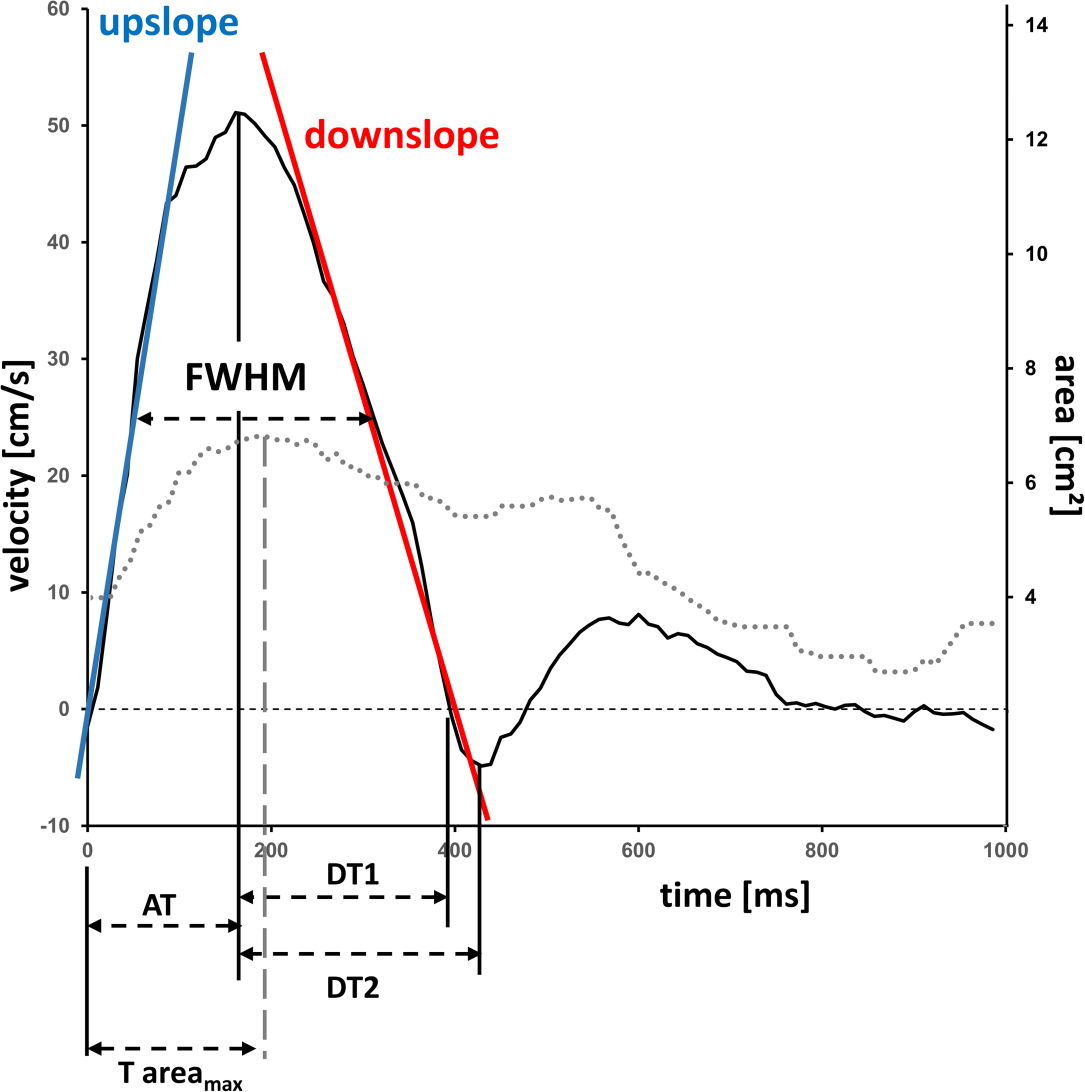
Velocity-time and area-time curve in the pulmonary artery by flow-sensitive CMR. Typical velocity-time curve (solid line) and area-time curve (dotted line) in the main pulmonary artery of a 17.9 years-old female participant. Calculation of vessel characteristics such as upslope, downslope, vessel areas and full width half maximum. AT, time-to-maximum velocity; DT1, deceleration time maximum-to-zero crossing; DT2, deceleration time maximum-to-minimum; FWHM, full width half maximum, T area_max_, time-to-maximum area.

The following measures were assessed: effective and indexed total stroke volume (SV), peak velocity for PA pressure drop calculation using the modified Bernoulli equation $\left(\Delta P_{\mathrm{peak}}=4V_{\mathrm{peak}}^{2}\right)$ (17, 18), mean PA velocity, cardiac index and PA/AO ratio defined as maximum PA vessel area/maximum AO vessel area. Based on the pulmonary flow profile the maximum amplitude, upslope (defined as the slope of the data between 25% before and 25% after the FWHM crossing position on the left side of the flow curve), downslope (defined as the slope of the data between 25% before and 25% after the FWHM crossing position on the right side of the flow curve), pulse wave velocity (PWV), relative area change (RAC), and the full width half maximum (FWHM) were assessed (Figure 1). The tricuspid regurgitation fraction represents the difference between the right ventricular SV and the pulmonary effective SV.

PA pressure parameters were determined based on cardiac output according to literature (3, 19, 20), and are summarized below:

$$\begin{array}{c} \;\mathrm{mPAP}(\mathrm{mmHg})=1.37\;\frac{\mathrm{mmHg}}{L/\min}\times \mathrm{AO}\;\mathrm{cardiac}\;\mathrm{output}\frac{L}{\min}\\ \;+8.2\;\mathrm{mmHg}\;\\ \;\mathrm{sPAP}(\mathrm{mmHg})\;\;\;\;=1.5\times \mathrm{mPAP}+0.46\;\\ \;\mathrm{dPAP}(\mathrm{mmHg})\;\;\;=0.71\times \mathrm{mPAP}\text{–}0.66\;\\ \;\mathrm{PAPP}(\mathrm{mmHg})\;\;\;=\mathrm{sPAP}\text{–}\mathrm{dPAP}=0.79\times \mathrm{mPAP}+1.12\; \end{array}$$

with mPAP = mean PA pressure, sPAP = systolic PA pressure, dPAP = diastolic PA pressure and PAPP = pulmonary proportional pulse pressure.

PA stiffness parameter included the following parameters as described elsewhere (1, 20):

$$\begin{array}{cc} \mathrm{RAC}(\text{\%})\; & \;=(\mathrm{are}a_{\max}-\mathrm{are}a_{\min})/\;\\ & \;\mathrm{are}a_{\max}\times 100\;\\ \mathrm{PA}\;\mathrm{elasticity}(\text{\%})\; & \;=(\mathrm{are}a_{\max}-\mathrm{are}a_{\min})/\;\\ & \;\mathrm{are}a_{\min}\times 100\;\\ \mathrm{PA}\;\mathrm{distensibility}(\text{\%}/\mathrm{mmHg})\; & \;=\mathrm{PA}\;\mathrm{elasticity}/\;\\ & \;\left(\mathrm{sPAP}-\mathrm{dPAP}\right)\;\\ \mathrm{PA}\;\mathrm{compliance}(mm^{2}/\mathrm{mmHg})\; & \;=(\mathrm{are}a_{\max}-\mathrm{are}a_{\min})/\;\\ & \;\mathrm{PAP}P^{*)}\;\\ \mathrm{PA}\;\mathrm{capacitance}\;(mm^{3}/\mathrm{mmHg})\; & \;=\mathrm{right}\;\mathrm{ventricular}\;\mathrm{SV}/\mathrm{PAP}P^{*)}\;\\ \mathrm{Elastic}\;\mathrm{modulus}\;(\mathrm{mmHg})\; & \;=\mathrm{PAP}P^{*)}\times \mathrm{are}a_{\min}/\;\\ & \;\left(\mathrm{are}a_{\max}-\mathrm{are}a_{\min}\right)\;\\ \begin{array}{c} \;\mathrm{Stiffness}\;\mathrm{index}\;\mathrm{betaindexed}\;\;\\ \;\mathrm{pulmonary}\;\mathrm{vascular}\; \end{array}\; & \;=\ln(\mathrm{sPAP}/\mathrm{dPAP})/\mathrm{PA}\;\mathrm{elasticity}\times 100\;\\ \;\mathrm{resistance}\;(\mathrm{PV}R_{i})(\mathrm{Wood}\;\mathrm{units}/m^{2})\; & \;=(\mathrm{mPAP}-\mathrm{PCWP})/\;\\ & \;\mathrm{PA}\;\mathrm{cardiac}\;\mathrm{index}\;\\ \;\mathrm{pulse}\;\mathrm{wave}\;\mathrm{velocity}\;(m/s)\; & \;=\Delta Q/\Delta A;\; \end{array}$$

calculated according to literature based on the *QA* (flow/area) method (21, 22). Values between 50 and 110 ms (early systole) were used to evaluate the slope of flow- area data. *) PAPP was used as PA pulse pressure in the formulas according to Sanz et al. (20).

Pulmonary capillary wedge pressure (PCWP) is calculated by CMR using the Nagueh formula [PCWP = 1.9 + (1.24 × *E*/*É*)] (23), where *E* is the maximum velocity of the *E*-wave of the transmitral flow measurement and *É* is the average of the septal and lateral early diastolic mitral annular tissue velocities. The determination of early diastolic mitral annular tissue velocity was done as follows: Locating the significant opening of the mitral valve. Measurement of the two distances between the lateral annulus to the apex and the septal annulus to the apex (=first measurement point). Repeating the same measurements 2 or 3 heart phases later (depending on the heart rate), corresponding to 50–60 ms (=second measurement point). The corresponding time difference between the two measuring points was calculated using the appropriate trigger delay tags from the DICOM header of the pulse sequence to determine the tissue velocity.

### 2.3. Statistics

Statistical analysis was conducted using SPSS (version 27.0.0.0, IBM Deutschland GmbH). Normal distribution was interpreted by the Shapiro-Wilk test. Continuous variables were presented as mean ± standard deviation (SD) if normally distributed, otherwise as median with interquartile range. Comparison of baseline characteristics, cardiac function and PA stiffness parameters with sexes or age groups was performed using unpaired Student's *t*-test for normally distributed data and Mann–Whitney *U*-test for non-normally distributed data. After checking the prerequisites for a linear regression analysis, such as linearity of the data, checking for outliers using boxplots and normal distribution, a linear Pearson product-moment correlation or a Spearman's Rho correlation analysis was performed to identify possible relationships between stiffness indices and age. Further correlations were calculated for PWV, RAC and Δ*P*_peak_ with various relevant parameters. Interpretation of correlation coefficients according to Cohen (24). *P*-values of <0.05 were considered statistically significant.

## 3. Results

### 3.1. Gender-specific characteristics of cardiac function

One-hundred-seventy-nine healthy volunteers were enrolled in this study ages 11–68 years with a median age [interquartile range] of 32.6 years [21.4;49.6]. Eighty-one subjects (45%) were male. Males exhibited greater PA maximal velocity (95 cm/s [85;105] vs. 80 cm/s [75;90]; *P* < 0.001) than females. Indexed RV and left atrial (LA) end-diastolic and end-systolic volumes were significantly increased in males. RV ejection fraction was significantly higher in females (62.7% [56.8;64.9] vs. 58.2% [54.8;62.2]; *P* < 0.001). No differences in PA cardiac index, AO cardiac index, global longitudinal, circumferential and radial strains were observed between genders. Further details are summarized in Table 1.

**Table 1:** Gender-specific subject characteristics and cardiac function.

	All	Male	Female	*P*-value
*N*	179	81	98	
Age (years)	32.6 [21.4; 49.6]	31.7 [20.5; 49.6]	34.3 [22.4; 48.6]	0.681*
Body surface area (m^2^)	1.84 ± 0.25	2.02 ± 0.21	1.69 ± 0.16	**<0**.**001**
PA heart rate (bpm)	67 [60; 73]	65 [60.0; 74.0]	68 [60.5; 72.5]	0.285*
PA effective stroke volume (ml)	91.5 ± 18.7	103.3 ± 17.1	82.5 ± 14.6	**<0**.**001**
PA total stroke volume, indexed (ml/m^2^)	50.6 ± 7.1	52.5 ± 6.9	49.5 ± 7.1	**0**.**008**
PA peak velocity [cm/s]	85 [80; 100]	95 [85; 105]	80 [75; 90]	**<0**.001*
PA cardiac index (L/min/m^2^)	3.30 [2.90; 3.70]	3.40 [2.90; 3.80]	3.29 ± 0.55	0.531*
AO cardiac index (L/min/m^2^)	3.10 [2.70; 3.50]	3.22 ± 0.64	3.09 ± 0.55	0.147
RV end-diastolic volume, indexed (ml/m^2^)	79.7 ± 12.3	87.0 ± 10.1	74.3 ± 11.4	**<0**.**001**
RV end-systolic volume, indexed (ml/m^2^)	31.0 [25.8; 36.9]	35.6 ± 6.7	27.8 [23.8; 31.8]	**<0**.001*
RV stroke volume, indexed (ml/m^2^)	47.9 ± 7.2	50.8 ± 6.7	45.5 ± 6.7	**<0**.**001**
RV ejection fraction (%)	60.2 [55.7; 64.0]	58.2 [54.8; 62.2]	62.7 [56.8; 64.9]	**<0**.001*
Tricuspid regurgitation (ml)	−2.84 ± 9.94	0.12 ± 11.5	−5.3 ± 7.7	**<0**.**001**
LA end-diastolic volume, indexed (ml/m^2^)	46.8 [40.9; 54.1]	50.2 ± 9.9	45.4 [39.7; 51.6]	**0**.003*
LA end-systolic volume, indexed (ml/m^2^)	20.6 [17.6; 24.7]	21.8 [18.5; 26.9]	20.1 [17.4; 23.4]	**0**.007*
LA ejection fraction (%)	55 ± 5	55 ± 6	56 ± 5	0.269
RV global longitudinal strain (%)	−18.9 ± 4.60	−18.5 ± 4.0	−19.1 ± 5.1	0.526
RV global circumferential strain (%)	−10.6 [−13.1; −8.5]	−10.5 [−13.1; −8.7]	−10.6 [−13.1; −8.3]	0.782*
RV global radial strain (%)	17.4 [13.8; 22.0]	17.8 ± 5.6	18.0 [13.3; 22.2]	0.960*

Data reported as mean ± standard deviation or median [interquartile range].

*n*, number of subjects; PA, pulmonary artery; AO, ascending aorta; RV, right ventricle; LA, left ventricle.

* Mann–Whitney-*U*-test otherwise unpaired Student's *t*-test.

Significant values are shown in bold.

### 3.2. Gender-specific characteristics of PA flow, PA velocity-time profile and PA stiffness indices

Males in contrast to females exhibited greater PA maximal velocity (*P* < 0.001), along with significantly higher levels of upslope (*P* = 0.007), downslope (*P* < 0.001), PWV (*P* = 0.018) and PA pressure drop Δ*P*_peak_ (*P* < 0.001). FWHM was significantly reduced for males versus females (*P* < 0.001). An exemplary illustration of the velocity-time profile is presented by Figure 1. PA elasticity (*P* = 0.096) and PA distensibility (*P* = 0.785) were comparable between both genders, whereas PA compliance (*P* = 0.033) and PA capacitance (*P* < 0.001) were higher in men. Mean, systolic and diastolic PAP were greater in males than females (all *P* < 0.001). Wedge pressure (PCWP) and PA/AO ratio between both sexes were not significantly different. Further details are summarized in Table 2.

**Table 2:** Gender-specific pulmonary artery flow, velocity-time profile and stiffness characteristics.

	All	Male	Female	*P*-value
Maximum amplitude (cm/s)	54.1 ± 10.5	58.7 ± 10.2	50.5 ± 9.3	**<0**.**001**
Upslope (cm/s^2^)*10^−3^	0.62 [0.51; 0.78]	0.67 [0.55; 0.80]	0.57 [0.50; 0.71]	**0**.007*
Downslope (cm/s^2^)*10^−3^	−0.24 [−0.30; −0.20]	−0.30 ± 0.08	−0.22 [−0.26; −0.18]	**<0**.001*
Pulse wave velocity (m/s)	1.83 [1.28; 2.70]	2.00 [1.35; 2.87]	1.73 [1.19; 2.34]	**0**.018*
Full width half maximum (ms)	228 ± 24	219 ± 22	235 ± 23	**<0**.**001**
Δ*P*_peak_ (mmHg)	2.9 [2.6; 4.0]	3.6 [2.9; 4.4]	2.6 [2.3; 3.2]	**<0**.001*
Relative area change (%)	36.3 ± 9.9	37.8 ± 9.7	35.0 ± 10.1	0.064
Mean PA velocity (cm/s)	14.6 [12.9; 16.6]	15.2 ± 3.8	14.3 [13.0; 15.9]	0.216*
PA/AO ratio (units)	1.07 ± 0.23	1.08 ± 0.23	1.06 ± 0.24	0.614
PA elasticity (%)	57.0 [40.7; 78.7]	60.4 [45.2; 82.2]	55.0 [37.7; 73.5]	0.096*
PA distensibility (%/mmHg)	4.22 [2.99; 5.54]	4.17 [3.07; 5.47]	4.26 [2.90; 5.65]	0.785*
PA compliance (mm^2^/mmHg)	19.5 [15.1; 24.7]	20.8 [16.6; 25.8]	19.2 ± 7.1	**0**.033*
PA capacitance (ml/mmHg)	6.6 ± 1.1	7.0 ± 1.0	6.2 ± 1.0	**<0**.001*
PA elastic modulus (mmHg)	24.0 [18.0; 33.0]	24.0 [18.0; 32.5]	23.0 [18.0; 34.5]	0.883*
PA beta stiffness index	1.45 [1.06; 2.04]	1.38 [1.00; 1.83]	1.53 [1.13; 2.24]	0.073*
TAPSE (cm)	2.24 [2.02; 2.53]	2.27 ± 0.38	2.27 [2.03; 2.54]	0.553*
Pulmonary vascular resistance index (WU/m^2^)	0.79 [0.67; 0.96]	0.74 ± 0.20	0.87 [0.72; 1.04]	**<0**.001*
PCWP (mmHg)	7.0 [6.2; 8.1]	6.8 [6.0; 7.7]	7.2 [6.4; 8.2]	0.064*
Mean PAP (mmHg)	16.0 [14.8; 17.2]	17.1 ± 1.8	15.3 ± 1.4	**<0**.**001**
Systolic PAP (mmHg)	24.5 [22.7; 26.2]	26.1 ± 2.7	23.5 ± 2.0	**<0**.**001**
Diastolic PAP (mmHg)	10.7 [9.9; 11.5]	11.5 ± 1.3	10.2 ± 1.0	**<0**.**001**
PAPP (mmHg)	13.8 [12.8; 14.7]	14.6 ± 1.4	13.2 ± 1.1	**<0**.**001**

Data reported as mean ± standard deviation or median [interquartile range].

PA, pulmonary artery; AO, ascending aorta; TAPSE, tricuspidal annulus plain systolic excursion; PCWP, pulmonary capillary wedge pressure; Δ*P*_peak_, pulmonary artery pressure drop calculated by using the Bernoulli formula; PAP, pulmonary artery pressure; PAPP, pulmonary artery proportional pulse pressure; WU, wood unit.

* Mann-Whitney-*U*-test otherwise unpaired Student's *t*-test.

Significant values are shown in bold.

### 3.3. Aging related PA flow, PA velocity-time profile and PA stiffness indices

**Figure 2:**
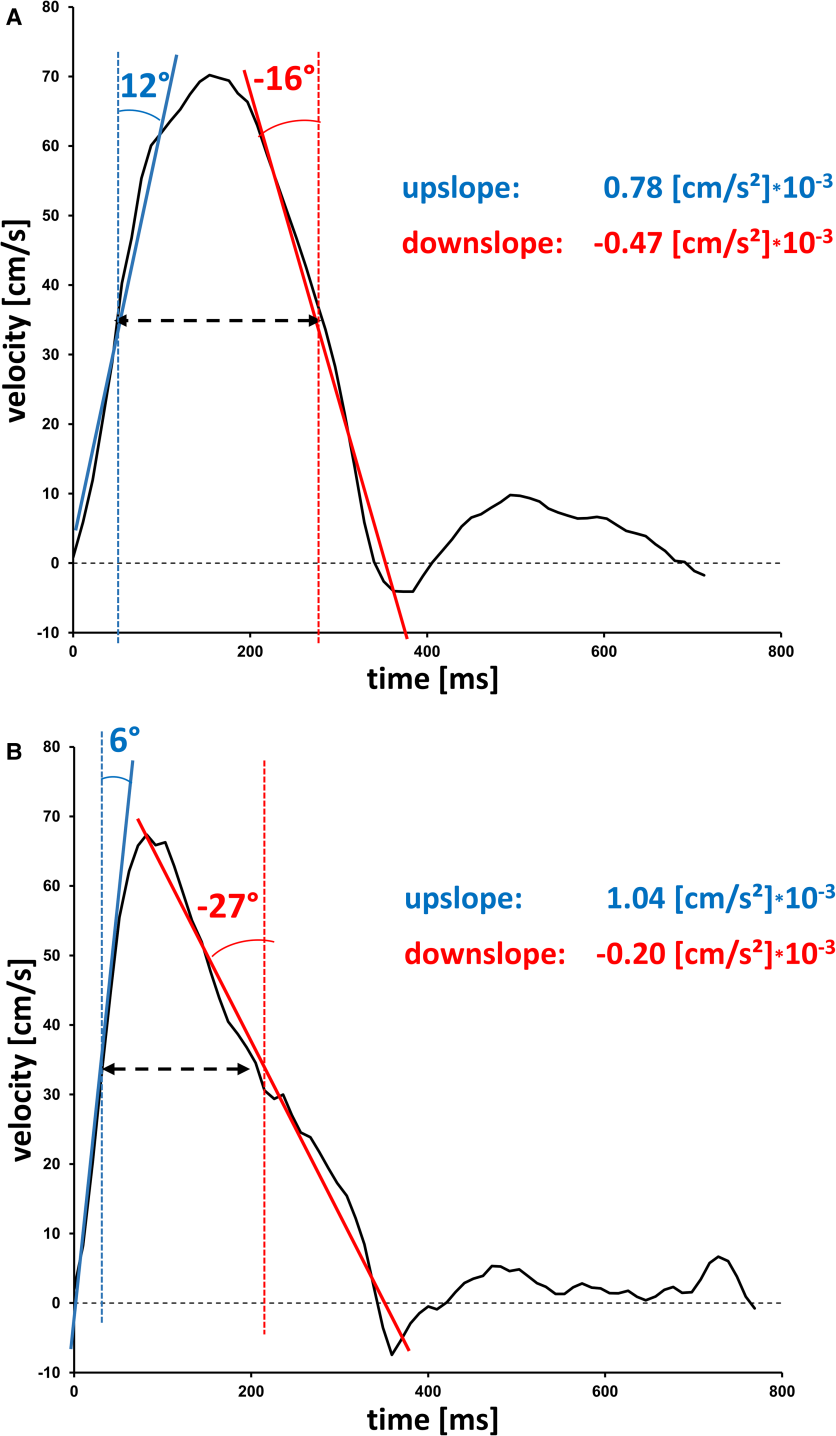
Age-dependent velocity-time curves. Velocity-time curves in the main pulmonary artery from (**A**) a young participant (female, 18 years) and (**B**) an older participant (female, 63 years). Typically, upslopes are larger in older subjects, whereas downslopes are smaller. Angulations are presented as a surrogate for slopes values.

Forty-three subjects (24%) were over the age of 50 years whereas 136 subjects were <50 years. The older in contrast to younger subjects presented significantly reduced PA elasticity, PA distensibility and PA compliance (all *P* < 0.001). These findings were accompanied by alterations in the velocity time profiles (Figure 2) characterized by significantly elevated upslope (*P* < 0.017), reduced downslope (*P* = 0.004), reduced FWHM (*P* = 0.001), and increased difference between the time-to-maximum area and time-to-maximum velocity (Δtime, *P* < 0.001) for subjects over 50 years of age. The PA/AO ratio was greater for young in contrast to the older volunteers (*P* < 0.001). Further details are summarized in Table 3.

**Table 3:** Pulmonary artery flow and velocity-time profile, geometry and stiffness characteristics in the age divided cohort.

	Age < 50 years	Age > 50 years	*P*-value
*n*	136	43	
Male (%)	44.9	46.5	
PA heart rate (bpm)	67.5 [60.0; 74.8]	65.1 ± 8.7	0.123*
PA total stroke volume, indexed (ml/m^2^)	51.6 ± 6.8	47.6 ± 7.1	**0**.**001**
PA maximal velocity (cm/s)	85.0 [80.0; 99.5]	90.0 [80.0; 100.0]	0.402*
Δ*P*_peak_ (mmHg)	2.9 [2.6; 4.0]	3.2 [2.6; 4.0]	0.396*
PA cardiac index (L/min/m^2^)	3.40 [3.00; 3.80]	3.00 ± 0.54	**<0**.001*
PA maximal area, indexed (*10^−4^)	4.28 ± 0.69	4.05 [3.40; 4.60]	0.173*
PA minimal area, indexed (*10^−4^)	2.60 [2.20; 3.00]	2.75 [2.40; 3.15]	0.081*
RV ejection fraction (%)	60.1 ± 5.3	61.2 ± 5.8	0.265
tricuspid regurgitation (ml)	−3.5 ± 10.1	−0.7 ± 9.1	0.105
LA ejection fraction (%)	56.1 ± 4.8	52.7 ± 5.5	**<0**.**001**
Time-to-maximum area (*T*_area_) (ms)	175 ± 49	206 ± 50	**<0**.**001**
Time-to-maximum velocity (AT) (ms)	133 [120; 148]	120 [102; 136]	**0**.005*
Δtime (*T*_area_—AT) (ms)	39 [1; 75]	84 ± 59	**<0**.001*
Upslope (cm/s^2^)*10^−3^	0.61 [0.51; 0.72]	0.72 ± 0.22	**0**.017*
Downslope (cm/s^2^)*10^−3^	−0.25 [−0.32; −0.20]	−0.22 ± 0.07	**0**.004*
Pulse wave velocity (m/s)	1.76 [1.24; 2.34]	2.59 [1.57; 3.59]	**<0**.001*
Full width half maximum (ms)	231 ± 21	218 ± 29	**0**.**001**
PCWP (mmHg)	7.0 [6.0; 7.9]	7.0 [6.3; 8.7]	0.220*
Mean PAP (mmHg)	16.2 [15.1; 17.4]	15.4 ± 1.7	**0**.005*
Systolic PAP (mmHg)	24.8 [23.1; 26.5]	23.6 ± 2.5	**0**.005*
Diastolic PAP (mmHg)	10.9 [10.1; 11.7]	10.3 ± 1.2	**0**.005*
PAPP (mmHg)	13.9 [13.0; 14.8]	13.3 ± 1.3	**0**.005*
Relative area change (%)	38.2 ± 9.9	30.1 ± 7.3	**<0**.**001**
Mean PA velocity (cm/s)	14.7 [13.1; 16.8]	14.1 ± 3.3	0.219*
PA/AO ratio (units)	1.11 [1.00; 1.24]	0.84 ± 0.18	**<0**.001*
PA elasticity (%)	66.4 [47.7; 83.0]	41.7 [31.0; 52.9]	**<0**.001*
PA distensibility (%/mmHg)	4.60 [3.29; 5.79]	3.38 ± 1.21	**<0**.001*
PA compliance (mm^2^/mmHg)	21.3 ± 6.8	15.4 [12.3; 20.7]	**<0**.001*
PA capacitance (ml/mmHg)	6.6 ± 1.0	6.5 ± 1.2	0.717
PA elastic modulus (mmHg)	22.0 [17.0; 30.0]	30.5 [24.8; 37.3]	**<0**.001*
PA beta stiffness index	1.26 [0.99; 1.74]	2.10 ± 0.74	**<0**.001*
TAPSE (cm)	2.25 ± 0.37	2.42 ± 0.46	**0**.026*
Pulmonary vascular resistance index (WU/m^2^)	0.80 [0.67; 0.98]	0.75 [0.62; 0.92]	0.190*

Data reported as mean ± standard deviation or median [interquartile range].

n, number of subjects; PA, pulmonary artery; AO, ascending aorta; AT, time-to-maximum velocity; RV, right ventricle; LA, left ventricle; TAPSE, tricuspidal annulus plain systolic excursion; PCWP, pulmonary capillary wedge pressure; Δ*P*_peak_, pulmonary artery pressure drop calculated by using the Bernoulli formula; PAP, pulmonary artery pressure; WU, wood unit.

* Mann–Whitney-*U*-test otherwise unpaired Student's *t*-test.

Significant values are shown in bold.

### 3.4. Correlation between aging vs. PA flow, PA velocity-time profile and PA stiffness indices

**Figure 3:**
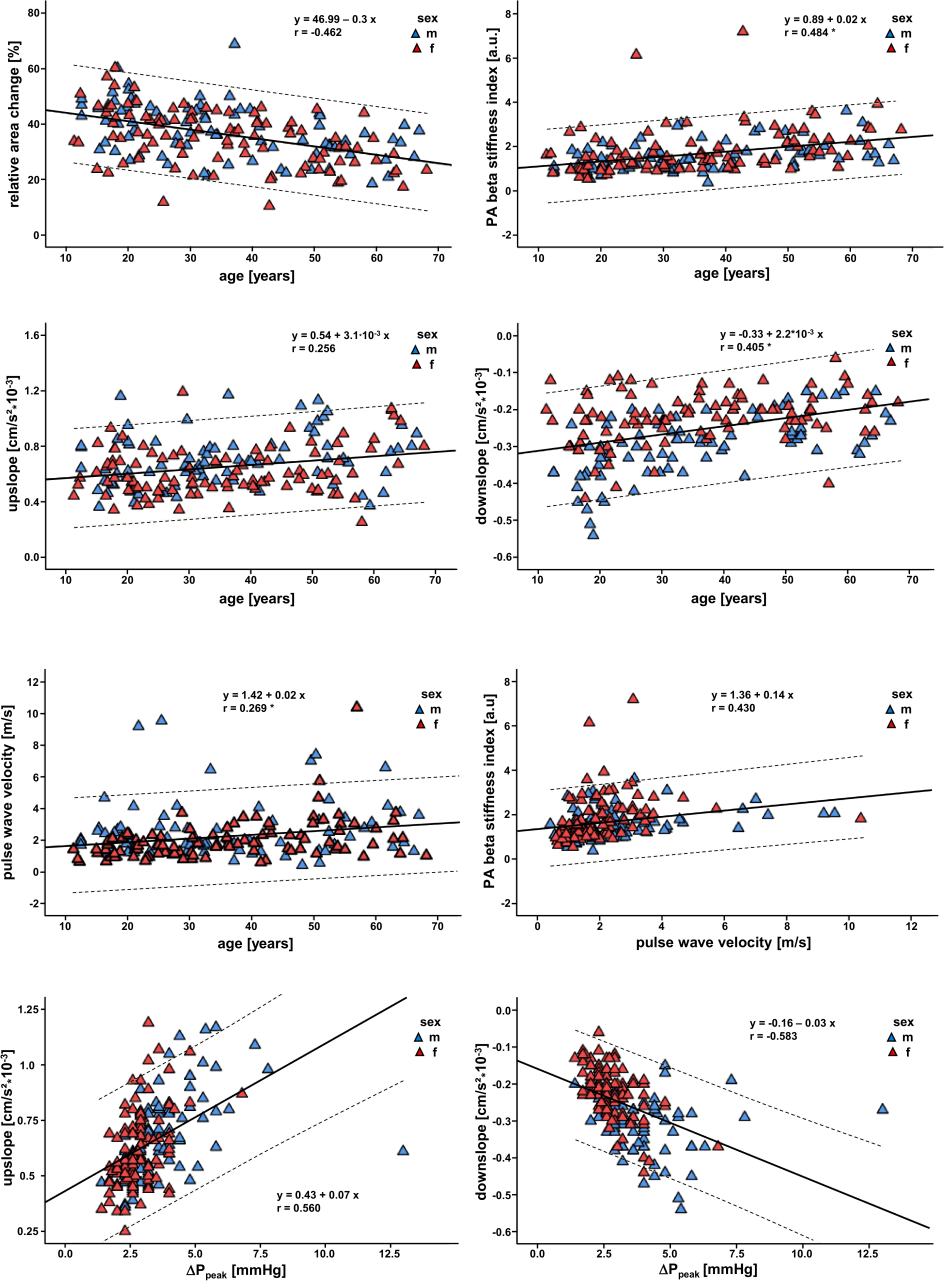
Correlations between the different parameters for assessing pulmonary artery stiffness. Scatter plots illustrating the linear correlations including 95% confidence intervals between different parameters of PA stiffness and age. *, Spearman's Rho correlation, Δ*P*_Peak_, pulmonary artery pressure drop.

Strong/moderate negative correlations were observed between PA elasticity, PA distensibility, PA/AO ratio, RAC, FWHM, Δtime (all *P* < 0.001) and aging. Additionally, upslope, downslope and PWV (all *P* < 0.001) correlated positively with age. No correlation was observed between Δ*P*_peak_, PA maximal velocity, RV ejection fraction and aging. Further details are summarized in Table 4. Exemplary correlations are illustrated in Figure 3.

**Table 4:** Correlations between PA flow, velocity-time profile and stiffness characteristics of the healthy cohort and age.

	r resp. rho	*P*-value	Cohen interpretation^a^
PA heart rate	−0.199	**0**.**007**	Weak
PA total stroke volume, indexed	−0.236	**0**.001*	Weak
PA maximal velocity	−0.088	0.242*	–
Δ*P*_peak_	−0.086	0.250*	–
PA cardiac index	−0.383	**<0**.**001**	Moderate
RV ejection fraction	0.043	0.568	–
Tricuspid regurgitation	0.047	0.536*	–
LA ejection fraction	−0.358	**<0**.**001**	Moderate
Δtime (*T*_area_—AT)	0.348	**<0**.**001**	Moderate
Upslope	0.256	**0**.**001**	Weak
Downslope	0.405	**<0**.001*	Moderate
Pulse wave velocity	0.269	**<0**.001*	Weak
Full width half maximum	−0.309	**<0**.001*	Moderate
PCWP	0.232	**0**.002*	Weak
Mean PAP	−0.248	**0**.**001**	Weak
Relative area change	−0.462	**<0**.**001**	Moderate
Mean PA velocity	−0.192	**0**.010*	Weak
PA/AO ratio	−0.577	**<0**.001*	**Strong**
PA elasticity	−0.481	**<0**.001*	Moderate
PA distensibility	−0.421	**<0**.001*	Moderate
PA compliance	−0.247	**0**.**001**	Weak
PA capacitance	0.095	0.204	–
PA elastic modulus	0.424	**<0**.001*	Moderate
PA beta stiffness index	0.484	**<0**.001*	Moderate
TAPSE	0.162	**0**.**031**	Weak
Pulmonary vascular resistance index	−0.313	**<0**.001*	Moderate

PA, pulmonary artery; AO, ascending aorta; AT, time-to-maximum velocity; RV, right ventricle; LA, left ventricle; TAPSE, tricuspidal annulus plain systolic excursion; PCWP, pulmonary capillary wedge pressure; ΔP_peak_, pulmonary artery pressure drop calculated by using the Bernoulli formula; PAP, pulmonary artery pressure; WU, wood unit.

^a^ Interpretation of r or Rho according to Cohen (24).

* Spearman's Rho correlation otherwise Pearson product-moment correlation.

Significant values are shown in bold.

### 3.5. Correlations between parameters of PA flow, RV function and PA stiffness indices

PWV correlates negatively with downslope (*P* = 0.003), FWHM (*P* = 0.004), PA elasticity (*P* < 0.001) and PA distensibility (*P* < 0.001), whereas PWV correlates positively with PA beta stiffness index (*P* < 0.001) and Δ*P*_peak_ (*P* = 0.006). RAC correlates positively with RV global circumferential strain (*P* < 0.009) and FWHM (*P* = 0.004). RAC correlates negatively with PWV (*P* < 0.001), and RV global radial strain (*P* < 0.010). There is a strong positive correlation between Δ*P*_peak_ and mean PAP, upslope, and downslope (all *P* < 0.001). Further correlations are illustrated in Table 5.

**Table 5:** Correlations between various parameters of PA flow, right ventricular function, and PA stiffness.

	r resp. rho	*P*-value	Cohen interpretation^a^
PWV vs. downslope	−0.222	**0**.**003**	Weak
PWV vs. FWHM	−0.213	**0**.**004**	Weak
PWV vs. Δ*P*_peak_	0.204	**0**.**006**	Weak
PWV vs. PA elasticity	−0.434	**<0**.**001**	Moderate
PWV vs. PA distensibility	−0.433	**<0**.**001**	Moderate
PWV vs. PA beta stiffness index	0.430	**<0**.**001**	Moderate
PWV vs. PVR_i_	−0.229	**0**.**002**	Weak
RAC vs. PWV	−0.434	**<0**.**001**	Moderate
RAC vs. FWHM	0.215	**0**.**004**	Weak
RAC vs. RV global circumferential strain	0.204	**0**.**009**	Weak
RAC vs. RV global radial strain	−0.199	**0**.**010**	Weak
RAC vs. RV end-diastolic volume, indexed	0.177	**0**.**018**	Weak
Δ*P*_peak_ vs. mean PAP	0.501	**<0**.**001**	**Strong**
Δ*P*_peak_ vs. FWHM	−0.373	**<0**.**001**	Moderate
Δ*P*_peak_ vs. upslope	0.560	**<0**.**001**	**Strong**
Δ*P*_peak_ vs. downslope	−0.583	**<0**.**001**	**Strong**
Δ*P*_peak_ vs. PVR_i_	−0.155	**0**.**038**	Weak

Only statistically significant correlations are shown.

FWHM, full width half maximum; PA, pulmonary artery; RV, right ventricle; PAP, pulmonary artery pressure; Δ*P*_peak_, pulmonary artery pressure drop calculated by using the Bernoulli formula; PWV, pulse wave velocity; PVR_i_, pulmonary vascular resistance index; RAC, relative area change.

^a^ Interpretation of r or Rho according to Cohen (24).

Significant values are shown in bold.

## 4. Discussion

**Figure 4:**
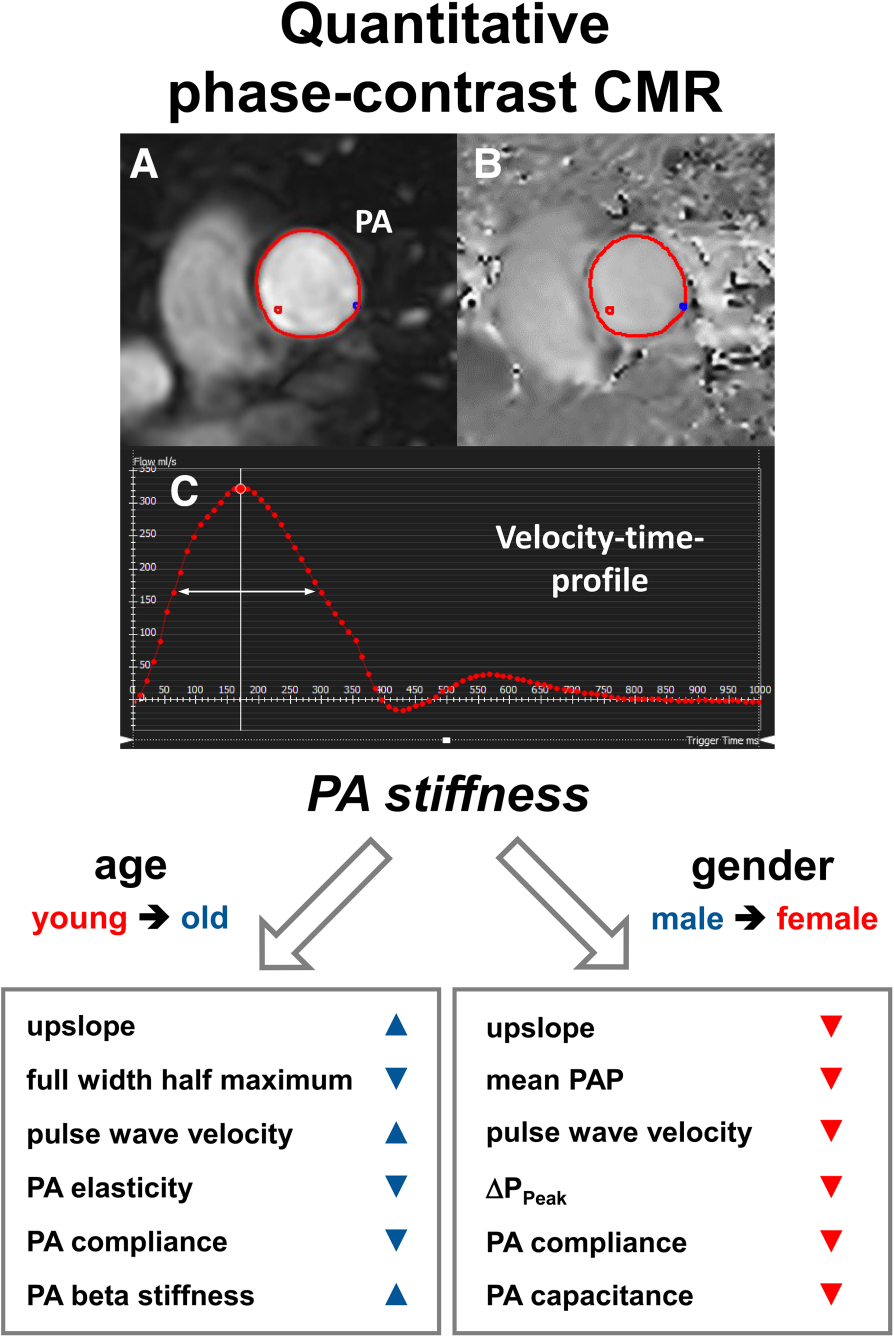
Assessment of PA stiffness measures by quantitative phase-contrast CMR. Male gender and older age are associated with increased pulmonary artery stiffness and altered velocity-time profiles. (**A**) Magnitude of phase-contrast CMR. (**B**) Phase of phase-contrast CMR. (**C**) Velocity-to-time profile curve. The white arrow indicates one of the important parameters (“full width half maximum”) for describing the stiffness properties. PA, pulmonary artery; PAP, pulmonary artery pressure, Δ*P*_Peak_, pulmonary artery pressure drop.

The present study provides a comprehensive CMR analysis of age- and gender-specific changes in PA flow, geometry, and stiffness for a large healthy cohort that, to our knowledge, has not been performed before (Figure 4). Particularly gender differences in PA hemodynamics remain clinically unestablished, but may explain epidemiological differences in disease development. The present CMR study demonstrates the following findings and novelties:

i) Provision of CMR acquired mean, systolic, diastolic PAP and PAPP for a healthy cohort respective of gender-differences.
ii) Males exhibit a higher basal level of Δ*P*_peak_, greater compliance and capacitance and a higher PWV than females.
iii) Aging is associated with a deterioration in PA stiffness indices, reduced RAC and a higher PWV.
iv) Velocity-to-time profile characteristics (upslope, downslope, FWHM and Δtime) change with age and gender.

### 4.1. Gender-associated changes in pulmonary hemodynamics

A novelty of this study is the provision of CMR-based mean, systolic, diastolic PAP and PAPP for healthy subjects. A comprehensive literature review had accumulated data for PA hemodynamics from 1,187 healthy individuals that received right heart catheterization. Their resting PA pressure appeared slightly reduced in contrast to our CMR-based measures. Per example, catheter acquired supine mPAP was 14.0 ± 3.3 mmHg (25) vs. our CMR-based mPAP of 16.0 mmHg [14.8;17.2]. However, the differences appear marginal, underscoring the potential of CMR as a surrogate for right heart catheterization.

Regarding gender-differences, males exhibit higher levels of all aspects of PAP compared to females. These findings were accompanied by greater compliance and capacitance of the pulmonary vasculature in males. We presume that the greater PWV and PA pressure in healthy males can be compensated with increased compliance and capacitance. Ultimately greater vessel distension during systole will result in elevated elastic recoil during diastole, as described by the Windkessel effect. Interestingly, measures of elasticity, beta stiffness index and distensibility remained comparable between both sexes, possibly due to insufficient sensitivity for detection of subclinical changes. These parameters are limited in the aspect of their strong parametric interdependence, suggesting that significant difference in one may be reflected in further parameters. However, the elevated PA pressures in males compared to females translated into a significantly increased maximum amplitude, upslope, downslope and reduced FWHM on the velocity-time curve. Although these hemodynamic differences in PA stiffness indices are not statistically detectable, they may indicate a higher baseline level of vascular stiffness within the pulmonary artery in men. As the majority of women were of pre-menopausal age, these differences may be due to the previously described vascular benefits of estrogen on arterial stiffness (26, 27) and the age-dependent decline in testosterone in males referred to as “andropause” (28). Overall, our results cannot explain why females are more susceptible to develop PAH. However, they support the observations that females exhibit more favorable hemodynamic profiles accompanied by superior RV function than males, translating into better survival rates (29).

### 4.2. Age-associated changes in pulmonary hemodynamics

In line with previous CMR observations for the pulmonary (14, 15) and the systemic (30) circulation, the multiparametric findings of the present study were suggestive of age-associated increased stiffness and decreased elasticity. Recent CMR studies of the pulmonary trunk described significantly increased PWV and decreased PA elasticity correlating with age. The present study identified a broader spectrum of parameters that define vascular aging of the pulmonary artery. Similar to gender-associated changes in the velocity-time curve, the upslope and downslope exhibited significant positive correlation with increasing age. Aging correlated with reductions in FWHM and RAC of the pulmonary artery. The markedly increased vessel stiffness, as reflected in the PA beta stiffness index, may explain the elevation in blood flow velocity and accelerated upslope. In contrast to the upslope, we found a flattening of the downslope among older subjects. This can be explained by the fact that after maximal vasodilatation has been reached, the regression of the vessel dimension may be delayed due to the reduced elasticity of the PA (see also Figure 2). Therefore, the flattening in the downslope curve is presumably linked to the reduction in PA elasticity, coinciding with significantly reduced PA distensibility and PA compliance. These assumptions are additionally supported by the considerably prolonged Δtime with increasing age, defined as the time difference between the time-to-maximum area and time-to-maximum velocity (*T*_area_ – AT). Due to the higher vascular elasticity in younger subjects, the time points for reaching the maximum blood flow velocity and the maximum vascular area are closer together. In contrast, the significantly increased stiffness index widens the interval between the two maxima. These findings were further consolidated by the correlative analyses, exhibiting a significant negative correlation between PA elasticity, PA distensibility, PA compliance and RAC with increasing age. Thus, vascular aging is associated with a lengthened time of elastic recoil. The complex interplay of molecular mechanisms contributing towards vascular aging have been extensively discussed (31).

The present study found a strong negative correlation between aging and the PA/AO area ratio. Due to breathing-associated alterations in diameter measurements for both PA and aortic diameter, we instead used maximal areas of both vessels to achieve greater consistency. The PA/AO ratio, however based on vessel diameters, was claimed to be an effective tool for detection of PAH patients (32) with a cut-off point >1.0. Boerrigter et al. had presented a normotensive patient group with mean age of 53.7 years to have an average PA/AO ratio of 0.87 in contrast to his PAH patients with 1.26 (32). However, this seems conflicting. Consistent with the results of a recent 4D flow CMR study (33), we observed a PA/AO ratio for the young (under 50 years) >1 whereas the older subjects (over 50 years) presented a PA/AO ratio <1. In line with Wehrum et al. (33), we find that PA area remains stable with age, whereas enlargement of aortic vessel area, possibly based on dilative arteriopathy, seems to be the cause of the ratio drop with increasing age. Therefore, it remains crucial to consider the age-associated dilative aortic remodeling that occurs in aging healthy subjects, to prevent the inclusion of false positive PAH patients.

Most interestingly, Δ*P*_peak_ is not found elevated for the older subjects (>50 years), compared with the younger subjects. Although we identify subclinical changes at a multiparametric level that describe vascular aging, the degree of vasculopathy remains limited, which explains the comparable Δ*P*_peak_ between young and old. One possible explanation may lie in concomitant cardiac remodeling, reflected by significantly reduced PA cardiac index and indexed PA total SV at constant RV ejection fraction. These observations are in line with recent study results (14). Therefore, in individuals >50 years of age, a reduced volume propulsion into the pulmonary system that has experienced aging-dependent loss of elastic and recoil function, may result in relatively constant PA pressure.

### 4.3. Correlations of PWV, RAC and Δ*P*_peak_ with various parameters

This study identified several powerful CMR-quantified correlations that, to the best of our knowledge, are reported for the first time for a healthy cohort. PWV and ΔP_peak_ (based on the Bernoulli formula) both show significant negative correlation with FWHM and downslope, due to reduced distension and elastic recoil of the vessel. This is further underlined by moderate negative correlations of PWV with PA elasticity, PA distensibility and PA beta stiffness index, although these do not share any parametric or algorithmic dependence on PWV. The inverse relationship between PWV and RAC was reflected in their considerable negative correlation. Impairment of elastic and distensible vessel function reduces the RAC, resulting in increased PWV, per example as a consequence of vascular aging. Comparable observations have been made in pathological state, per example, in patients with chronic obstructive pulmonary disease (34). Moreover, the impaired elasticity is associated with reduced temporal dimensions of the velocity time curve that are expressed by the strong positive correlation between RAC and FWHM. Moreover, RAC is coupled to RV function expressed by correlations with global radial and circumferential strains along with RV end-diastolic volume. This is to be expected, as RV filling, in combination with RV wall deformation, determines the blood volume propelled into the pulmonary vascular bed as a function of preload. With progression of vascular stiffness these cardiac hemodynamic alterations may result in structural remodeling of the heart associated with diastolic dysfunction. The observed correlations underscore the ability of CMR assessment in characterizing PA hemodynamics.

### 4.4. Limitations

This is a cross-sectional, single-center study and thus unable to determine causality. Subject selection was biased based on local population. Furthermore, the generalizability of the age-related results may be somewhat limited in the 50-plus age group because fewer individuals in this age group meet the cardiac healthiness criteria. On the other hand, we attempted to find optimal candidates >50 years of age for this study who met our stringent criteria for cardiac healthiness, and thus avoiding any significant bias. We present novel associations between age and gender with pulmonary hemodynamic parameters. Comparable measured values from right heart catheterization were not available, as the invasive procedure is not indicated in healthy subjects, thus making it ethically unsupportable. Collection of comparable data including right heart catheterization in patients with pulmonary hypertension is planned at our institution. No intermodal comparisons were carried out, per example, between echocardiography and CMR. However, the formula demonstrated in the methods section of our study derive from previous catheter and echocardiographic data. Although it is to be expected that CMR somewhat underestimates the pressure drop Δ*P*_peak_ compared to echocardiography because the measured peak velocities are lower due to averaging over a longer data acquisition period, we were able to show in this study that Δ*P*_peak_ correlates significantly with other pressure parameters such as mPAP etc., which are determined by using the cardiac output. Nonetheless, the clinical value of our sex- and age-dependent reference values require validation in larger cohort studies.

## 5. Conclusion

The observed differences in pulmonary stiffness indices, PA pressure parameters and overall hemodynamics, represented by velocity-to-time profile features such as upslope, downslope, FWHM and Δtime, are likely attributed to gender and aging. Male sex is associated with a higher basal PA pressure parameters and different velocity-time profiles. Older age is associated with reduced elasticity, increased stiffness and altered velocity-time profiles. CMR assessment shows effective screening potential for PA hemodynamics and can serve as a substitute for right heart catheterization.

## Data Availability

The raw data supporting the conclusions of this article will be made available by the authors, without undue reservation.
